# How to Minimize the Impact of Experts’ Non-rational Beliefs on Their Judgments on Autism

**DOI:** 10.1007/s10597-022-01062-1

**Published:** 2022-12-03

**Authors:** Maciej Wodziński, Marcin Rządeczka, Marcin Moskalewicz

**Affiliations:** 1 Institute of Philosophy, Maria Curie-Skłodowska University, M. Curie-Skłodowska Sq. 4, 20-031 Lublin, Poland; 2 Doctoral School of Humanities and Art, Maria Curie-Skłodowska University, Weteranów 18, 20-038 Lublin, Poland; 3 Philosophy of Mental Health Unit, Poznan University of Medical Sciences, Rokietnicka 7, 60-806 Poznan, Poland

**Keywords:** Autism, Expert knowledge, Debiasing strategies, Stereotypes, Non-rational beliefs

## Abstract

The non-autistic majority often judges people on the autism spectrum through the prism of numerous stereotypes, prejudices, cognitive biases, or, generally speaking, non-rational beliefs. This causes problems in autistic people’s everyday lives, as they often feel stigmatized, marginalized, and they internalize deficit-laden narratives about themselves. Unfortunately, experts, including health or law professionals, are not entirely immune to these non-rational beliefs, which affect their decision-making processes. This primarily happens when a mix of background knowledge, overconfidence, and haste co-occur. The resulting decisions may impact autistic people, e.g., by determining eligibility for the state’s therapeutical and financial support. This paper shows how simplified reasoning and inference may influence experts’ (medical examiners or court expert witnesses) decision-making processes concerning autistic people. It also proposes particular clues and strategies that could help experts cope with this risk and avoid making biased decisions.

## Introduction: Expert Knowledge and Decision-Making Processes

It is well known that people use stereotypes and simplified methods of inference (heuristics) when making judgments. It is also well known that they make systemic, predictable cognitive errors based on heuristics. Individuals commonly regarded as experts in their field are also subject to simplified rules of inference and stereotypes (Gigerenzer, 2015; Kahneman, 2013; Kahneman & Tversky, 1974; Kahneman et al., 1982; Meehl, 2013; Oskamp, 1965). Even domain-specific experts are not entirely immune to the influence of common stereotypes and simplified reasoning patterns (Draaisma, 2009; Hacking, 2007, 2010).

Two primary theories on the role of heuristics in expert decision-making processes differ by valuing expert mental shortcuts in opposing ways. According to the *Naturalistic Decision Making* theory, heuristics allows experts to make complex decisions in a very short time (Gigerenzer, 2015). For example, experiments showed high accuracy and effectiveness of chess players and firefighters (Kahneman & Klein, 2009; Klein, 1993; Klein et al., 2010). On the other hand, the *Heuristics and Biases Approach* theory values heuristics negatively as a significant source of expert cognitive errors (Kahneman et al., 1982). Both perspectives agree that heuristics is indispensable in our decision-making. We cannot turn it off. We may only try to control its effects. And heuristics can be very useful. For example, due to the minimal time available to examine a case, opinions given by experts in a case-law process must be based mainly on intuitive decisions (Chase et al., 1973; Kahneman, 2013; Simon, 1992).

In this regard, both theories also point out three primary conditions required for correct intuitive decisions (Damasio, 2006; Ericsson, 2008), which, as we shall argue, are not met in mental health assessments concerning autism.^1^ (1) Up-to-date knowledge based on credible sources in a given domain. (2) Deliberate practice, i.e., a long-term, systematic, and purposeful application of expertise continuously subjected to reflection. (3) Good quality feedback on the decisions made. Failure to meet these conditions exposes experts to mistakes and, more importantly, prevents them from eliminating these mistakes. It creates a vicious circle of wrong decisions strengthening and fixating the mistakes leading to further incorrect decisions.

Due to the nature of the evaluated phenomena, experts from domains such as psychiatry or psychology are particularly prone to committing systemic cognitive errors (Shanteau, 1992). In contrast to most of the other domains, mental states, disorders, or illnesses are difficult to measure in an objectified way. Experts must deal with the lack of biomarkers, big personal differences, non-specific symptoms, and co-existence of various conditions, including somatic ones. They also often base their judgments on intuitions whose reliability is unclear (Moskalewicz et al., 2021; Moskalewicz & Gozé, 2022).

The diagnosis of autism is particularly problematic due to the lack of unequivocal findings as to its causes. Its increasing popularity translates into vivid presence in the public sphere and often, inadvertently, to the rise of autism-related stereotypes. These stereotypes are the essential discursive preconditions of cognitive errors concerning autism, which is why we shall now give their brief overview.

The power dynamics between autistic people and clinical experts described in this article can be said to be only an exemplification of the wider problem of power relations between autistic and non-autistic people. These power relations impact almost every area of autistic people's lives, from the construction of their identities (based e.g. on social, media, and professional representations), through interpersonal relationships at all levels of social organization, to decisions made by third parties related to their economic and social situation.

This is all the more significant because the vast majority of these power relations are largely shaped by non-autistic people, while the much-needed first-person perspective of those most concerned is overlooked. This situation is fortunately changing, particularly through the work of self-advocates and many autism-related communities.

While aware of the relevance of the broad power dynamics between autistic and non-autistic people, in this paper we focus on the subset of power dynamics between autistic people and health professionals/experts. This is also because the space of this article is limited, and we aim to offer some concrete solutions to a particular problem. Moreover, the case of power dynamic presented in the paper describes quite representative example due to the fact that decisions made by health professionals have generally long-term, if not lifelong consequences, for their autistic patients. They heavily and directly influence their quality of life, self-esteem, stress burden, and, last but not least, relationships with non-autistic individuals.

## Public Views on Autism

According to professional diagnostic manuals autism is a neurodevelopmental condition often manifested in qualitative atypicalities in the areas of sensorial and information processing, social interaction, verbal and non-verbal communication, and thinking and behaviour patterns difficult to modify (American Psychiatric & Association, 2013). Not every autistic person, however, manifests atypicalities from all the areas, e.g. is extremely sensitive to sensorial stimuli and simultaneously communicates in a qualitatively different way. Due to significant increase in diagnosed cases autism has become an issue on a societal scale. In the 1950s, the incidence of autism was 1/10,000 people (including adults). In 2002, the American CDC reported 1 in 150 children; in 2012, 1 in 68, and in 2014, 1 in 59 children (Baio et al., 2018). Today, for every 10,000 people, not 1, but almost 170 may be autistic. Despite many years of research in various fields of medicine, psychology, and neuroscience, the exact causes of autism are not yet known. The hypotheses range from genetic (Robert et al., 2017), including intragenomic conflicts (Badcock & Crespi, 2006), gut-brain axis disorders (Diaz Heijtz et al., 2011; Montiel-Castro et al., 2013), developmental environment (Grabrucker, 2012; Karimi et al., 2017), vaccination (repeatedly discredited), and time processing disorders (Allman & DeLeonIser, 2008; Allman & Falter, 2015), to name a few.

Due to extensive social campaigning (screening programs, World Autism Awareness Day, UN classification of autism among the most serious health problems in the world) and tremendous interest from the media and scientific communities, autism is widely registered in social awareness. Therefore, the quality of its media representation exerts an important impact on both laypeople and professionals. It may also play a significant role in the process of self-identification of autistic people (Bagatell, 2007; Rourke & McGloin, 2019).

The image of autistic people often presented in the media correlates with their social perception (Draaisma, 2009; Hamilton & Krendl, 2007). Popular media such as television, the press, and the Internet especially, are usually entirely deprived of any control of the reliability of information. This often creates a one-sided and little nuanced image of autism and autistic people. Obviously, different media address different social needs and thus present their own version of autism.

The perception of autism established in the 1940s–50 s as a “severe disorder” (characterized by high support needs) that makes people unwilling or unable to communicate with others (something that Hacking defines as *core autism*) used to be present in film and television productions for years. Influential and highly renowned movies attracting large audiences, like Rain Man, Mercury Rising or K-Pax, portray autistic people as “amazing curiosities” with genius minds. They often appear as background characters for main protagonist and act as “tools” for solving specific problems. They also show no inner life nor self-development as a story unveils (Baker, 2007; Ejaz, 2020; Nordahl-Hansen et al., 2018).

Today's productions present a significant change. Usually, they present autistic people as individuals functioning in society with some difficulties. Some TV shows made in recent years, such as *The Good Doctor*, *Atypical* or *The Big Bang Theory* are examples of good practices in this area. Film or TV makers are more aware of the potential impact of their productions on social perception. Nowadays, when depicting autistic people, they turn to appropriate advisers, who ensure a reliable presentation of the condition and even attempt to dispel some prevailing stereotypes. The question of who should act as an “appropriate” advisor and represent the community remains. Nevertheless, these new productions are clearly created with greater awareness as they present first-person perspectives of autistic protagonists with rich inner and social lives. At the same time, unfortunately, these new productions still reproduce some stereotypes, such as those of age and gender (mainly showing male children or teenagers), specific kinds of behavior, genius mind or other kind of “superpower” (Garner et al., 2016; Young, 2012). In this sense, their impact on social perception of autism may be still full of oversimplifications and potentially harmful. Additionally, common stereotype negates the fact that autistic people as a group are extremely heterogeneous and often ‘not as special’ as they are expected to be by the non-autistic majority.

Press discourse could present an alternate case. Due to a different social role that newspapers play, one could expect a journalistic autism portrayal that is more nuanced, fact-based, diversified, and less “dramatic” than that of film makers. Unfortunately, many studies from different countries show that in many cases these expectations fail. Although the situation is getting better press discourse is still far from perfect. It still ignores the voice of autistic people (Bie & Tang, 2014; Huws & Jones, 2010), imposes the deficit perspective (Holton et al., 2012; Jones & Harwood, 2009), uses medical-centered and not social narration (Billawala & Wolbring, 2014; Wolbring & Mosig, 2017), and presents autism as a “medical problem” and not an alternative mode of experience (Jones & Harwood, 2009; McKeever, 2012).

It should be also noted that in various media autism is mainly portrayed as almost all white phenomenon, with main characters of the most popular tv shows and press articles being white males (Heilker, 2012; Onaiwu, 2020). The scientific community also seems to marginalize people of color in the research (Malone et al., 2022). Such homogenic autism representation might be especially disturbing as there is some evidence that race and ethnicity of an autistic child may influence the work of clinicians resulting in different thresholds for diagnosis or even substantially delay it (Becerra et al., 2014; Mandell et al., 2009).

Unfortunately, as several studies show, these stereotypical and deficit-laden narratives diffuse not only to broad social awareness, but also to experts’ thinking (Bargiela et al., 2016; Georgiou et al., 2021).

However, when information from the medical/expert field and the media intertwine, it is not difficult to make a mistake that becomes difficult to correct once it reaches public awareness. For example, despite the undeniable improvement of autistic people representation in the media, the results of a recent survey from 2018 (Polish population, n > 1 000) show that participants still associate the image of an autistic person with what is sometimes called a “core” autism with high support needs in many areas) (Omyła-Rudzka, 2018).

## Non-rational Beliefs About Autism

We shall call these untruthful and publicly spread ideas non-rational beliefs. We choose the term over stereotypes, for stereotypes must not be necessarily false. On the other hand, we prefer the term over false beliefs, for the latter is usually associated with delusional convictions. Non-rational beliefs are not delusional, for they are widespread in society among those lacking expert knowledge. At the same time, non-rational beliefs are more than stereotypes, for they always result in societal misunderstanding of the autism’s symptoms and genesis, harmful pseudo-medical practices, social stigma, and professional exclusion (Humphrey & Symes, 2010; Marsack & Perry, 2018; Richards, 2012).

Autistic people and their relatives face a variety of non-rational beliefs about themselves, both from the wider society, i.e. people without expertise, and worse from health professionals (Nicolaidis et al., 2015). For example, they have a fear of disclosing their diagnosis to health professionals. A study by Nicolaidis and colleagues on the interaction of autistic people with healthcare providers also showed people on the spectrum being considered as lacking empathy, not caring about the feelings of others, and even dangerous. To give an example statement from a study participant: "I break into tears at the drop of a hat. So what I've learned to do is to shut down. When I'm feeling empathy, I go 'Oh, no, no, you're gonna fall apart, so shut down now. Just shut down, because you don't want to look like you're a crazy woman. So shut down.' So instead of looking like a crazy woman … I look like a cold-hearted bitch' (p. 829).Equally problematic is the non-rational belief that all autistic people do not communicate verbally with others and display identical patterns of behavior, such as being completely withdrawn from interpersonal relationships. In a study conducted by Bargiela and colleagues, autistic women indicated that their counter-stereotypical behavior, such as possessing good social skills, impacted professionals' willingness for issuing the diagnosis (Bargiela et al., 2016).

Furthermore, the very widespread belief that autism predominantly occurs in children and in men, related, among other things, to the adaptation of diagnostic criteria to men's behavioural patterns and socially imposed behavioural camouflaging strategies in women during socialisation process (Haney, 2016), may result in adults and women and non-binary people false negative diagnosis. In addition, the medical professionals undermining of diagnosis already held leaves many autistic people without help and understanding in a world that is predominantly not adapted to their needs.

Thus, as Treweek and colleagues note it is “evident that societal stereotypes can lead to non-disclosure and delayed diagnosis, both of which may prevent autistic people accessing the services they are entitled to'. Moreover, all of the aforementioned non-rational beliefs can influence the so-called sense of self-illness ambiguity (Glas & Dings, 2020) and lead to a process of self-stigmatisation (Corrigan et al., 2016).

Numerous studies and first-person accounts of autistic people have shown that these beliefs are not true (Berthoz & Hill, 2005; Connor, 2000; Pisula et al., 2017; Shipman et al., 2011; Zamoscik et al., 2016). Still, they may have serious consequences for autistic people.

Only few studies focus on how the autistic individuals think about their social image. Interestingly, one of the most harmful yet unobvious non-rational beliefs about autistic individuals is the neglect of their self-awareness in the realm of social functioning. Common belief that the main challenge for autistic people is social functioning contrasts with self-reports that indicate informational and sensory processing as the main challenge of daily life. Autistic people are aware of the fact that the non-autistic majority think of them as ‘weird’ and, as a result, focus purely on their allegedly social difficulties (Treweek et al., 2019). However, in at least some individuals, the social realm can be directly or indirectly affected by the core information and sensory processing difficulties. Unfortunately, social difficulties are ‘taken less seriously’ than information processing atypicalities because they are often reduced *per analogiam* to ‘funny’ social awkwardness depicted in many types of pop culture. Even experts could easily fall prey to the reasoning that autistic individuals are unaware of their social image or that they are aware but they do not care about it. Not taking into account autistic peoples’ awareness of how they are perceived by others is a preliminary neglect of their person's subjectivity, which, in some cases could possibly lead to dehumanization (Table 1).

**Table 1:** Examples of non-rational beliefs about autism functioning in public awareness and their possible consequences

Non-rational belief	Possible consequences
Autistic people are unhappy, suffering, emotionless, they lack adequate eye-contact (therefore they are not willing to make and sustain interpersonal relationships)	Considered insensitive, cold, and reluctant to communicate; social isolation, hindered integration with peers or colleagues
Autistic people lack empathy	Emotional needs and states disregarded both in social relations and in health professional context
Women and non-binary people cannot be autistic	False negative diagnosis, difficulties in accepting diagnosis, hiding autistic traits
Autistic people do not speak and inhabit their own world	Autism diagnosis questioned by experts, refusal of social services’ support
Autism is only a childhood condition	False-negative diagnosis of adults, hiding autistic traits, limited social awareness of undiagnosed adults, lack of social support
Autism is caused by vaccinations	Home chelation treatments (intended to remove heavy metal ions from the body) that carry a major risk of side effects; noticeable decline in public vaccination rates that increase epidemiological threats
Autism is a communicable disease that can be cured once and for all	“Treatment” of autism with strongly bactericidal chlorine-based preparations (bleach) with severe health consequences (Trudeau et al., 2019); school segregation; depression of autistic children (Ghaziuddin et al., 2002; Kinnear et al., 2016; Stewart et al., 2006)
Autistic individuals are unaware of how they are socially perceived or do not care about it	Neglect of subjectivity and self-awareness, dehumanization

## From Laypeople to Expert Non-rational Beliefs

Some of these non-rational beliefs, such as the ‘treatment’ of autism by application of diluted bleaches, are a serious menace to health (Harrison & Zane, 2017). To effectively counteract them, we must first understand their cultural origins as well as schemes and simplifications of reasoning that lead to their wider acceptance. The bleach treatment belief emerged from credible research conducted since the early 2000s on the gut-brain axis. This concept assumes a negative influence of abnormal intestinal microbiota on the secretion of cholecystokinin (a group of peptide hormones secreted by duodenal mucosa) that plays a significant role in regulating many brain development and functional processes. Research on the impact of the gut-brain axis on the formation of autism has been conducted for years without unequivocal outcomes (Hosie et al., 2019; Li & Zhou, 2016; Yang et al., 2018). Some correlations between the occurrence of disturbed intestinal flora and autism symptoms have been found, but no real cause-and-effect relationships (Yang et al., 2018).

Assuming that only a small percentage of parents of autistic children or journalists writing about this topic will try to search for information in strictly scientific sources and take the trouble of analyzing research articles, most of them will end up checking sources that are readily available, such as online encyclopedias and parenting websites. For example, the Polish Wikipedia entry for *gut-brain axis* contains a direct statement that dysfunction of the axis is one of the *causes* of autism (Wikipedia., 2020). Only a closer examination of the entry’s sources shows that the scientific research considers microbiota disorders as only one of the possible factors affecting autism development. A layperson’s inference process may be quite straightforward. (1) Autism occurs and (2) prevailing therapies do not yield the desired effects, therefore (3) one should look for other solutions. Furthermore, since readily available sources of information based on scientific research indicate that improper intestinal tract microbiota leads to autism, one could, therefore, (4) try to remove the dangerous intestinal flora. Searching online for instructions on how to carry out this procedure, parents come across information about the possibility of administering a strongly bactericidal, effective, readily available, and inexpensive mixture—bleach. People sometimes get better after such treatment but the causal relationship is likely different. When possible digestive tract ailments are eliminated, a person’s willingness to get in contact with others would likely improve. Furthermore, such positive effects may obscure possible effects of simultaneously applied standard therapies. This does not mean that autism was cured. The potential benefits of such ‘treatment’ often overshadow the dramatic consequences of drinking diluted bleach. However, possibly due to the so-called focus effect (Connor, 2000), belief bias (Evans et al., 1983) or halo effect (Kahneman, 2013), these facts are ignored. The steps described above are usually taken by desperate people who are not satisfied with ordinary therapies, but they also result from the lack of hard and systematic therapeutic work with the child.

Non-specialists who do not possess professional background (such as parents of autistic children or journalists) succumb too quickly to *System 1* described by Daniel Kahneman (an “automatic”, intuitive and fast way of thinking, in contrast to System 2, which is slower, more analytical and algorithmic way of thinking and inferencing). In this particular case, they notice a causal relationship instead of a correlation and turn it into a conviction that the defective intestinal flora undoubtedly triggers autism. However, also experts (such as court expert witnesses or medical examiners) are prone to simplified modes of reasoning and may succumb to culturally prevalent non-rational beliefs/stereotypes on autism. As Alvin Goldman argues, the issue of expert knowledge and expertise in general is strictly connected with the broad context of social epistemology. Therefore, experts’ work is heavily entangled with the socially prevailing state of knowledge (Goldman, 2018). Moreover, in some atypical or mixed contexts experts may not be fully aware whether or not they are asked about their opinion as experts. Hence, they may use different epistemic standards and modes of reasoning. This fact may limit their ability to issue reliable opinions.

Shanteau (1992) shows that because of the high degree of uncertainty, psychologists and psychiatrists belong to a group of experts who are exposed to relatively frequent cognitive errors. In case of medical diagnoses, this may result in a 10–15% rate of mistakes in clinical diagnosis processes (Elstein & Higgs, 1995). In their 2005 review study of the relationship between clinical experience and quality of health care, Choudhry, Fletcher and Soumerai showed that physicians’ (including psychiatrists’) quality of clinical practice often declines in the long term (Choudhry et al., 2005). Therefore, expert opinions issued by medical examiners or court expert witnesses may contain errors, e.g. cognitive biases (O’Sullivan & Schofield, 2018). This is especially the case when despite their formal education experts lack sufficient, up-to-date knowledge or deliberate practice (Ericsson, 2008). According to the 2015 report of Helsinki Foundation for Human Rights, it is not as uncommon among court expert witnesses as one could suspect (Helsinki Foundation for Human Rights, 2015). Also, according to Treweek and colleagues (Treweek et al., 2019), who comment on Bargiela’s (Bargiela et al., 2016) qualitative research on autistic people, it is “evident that societal stereotypes can lead to non-disclosure and delayed diagnosis, both of which may prevent autistic people accessing the services they are entitled to.”

It is worth noting that some of the stereotypes indicated in research on social perception of autism (Holton et al., 2012; Huws & Jones, 2010; Jones & Harwood, 2009; Omyła-Rudzka, 2018; Wodziński & Gołaska-Ciesielska, 2021; Wolbring & Mosig, 2017), such as those regarding “being closed in one's own world,” “lack of interpersonal relationships,” or “avoiding eye contact,” may be also relevant to health professionals.

Individuals who do not exhibit the stereotypical characteristics of being cut off from the world may not be accurately diagnosed. The non-rational beliefs may also negatively affect applications for state funds or services by autistic people. When experts determine their level of functioning, the social image of autism plays an important role. It happens that experts question the need for state assistance and sometimes negate the previous diagnosis (Wodziński, 2020). Although this paper refers to the particular examples from Polish legal regulations and health assessment institutions, the problem of cognitive biases in experts’ decision making processes is a common problem on a global scale. The mechanisms responsible for cognitive biases are in large part universal and unrelated to nationality, ethnicity or language, as the example of UK Parliament’s Works and Pensions Committee inquiry about the quality of experts’ assessments for disability and health-related benefits shows.^2^

Furthermore, some of the experts’ opinions and decisions reflect the socially prevailing bio-medical view of autism (Billawala & Wolbring, 2014; Huws & Jones, 2010; Wolbring & Mosig, 2017). Even a brief analysis of their decisions—some of which, in the case of Poland, can be accessed through the Portal of Common Courts Rules—shows that self-sufficiency, which is one of the main criteria for granting state’s financial support, is often understood in terms of solely physical abilities (e.g., moving around). In the case of autism, however, physical and somatic difficulties do not prevail. It is mainly the sphere of social and emotional functioning or interpersonal communication that causes most of the self-sufficiency issues. Experts succumbing to the bio-medical perspective on autism may often not notice and understand that autistic people’s difficulties are mainly functional and not physical. This may lead to biased decisions, whose consequences are highly troublesome for autistic people, including cuts on the state's financial and therapeutic support.

To become a court expert in the Polish justice system, one has to “possess theoretical and practical special information in a given branch of science, technology, art, craft” (Polish Journal of Laws No. 15, item 133). In the case of medical appraisers, these requirements do not even specify the specialization that a doctor should have in order to evaluate specific cases. Such a wide and general definition of the competencies required to become a court expert does not give the courts that appoint these experts the proper tools to verify the actual competencies of a given person in a narrow domain of knowledge, for example in reference to autism. Moreover, the current legal regime has a huge problem with personnel shortages among medical appraisers, especially in child psychology, which results in the appointments of persons from other specializations or lacking appropriate experience (for example, a phoniatrist determines the disability level for an autistic person). This situation often leads to incompliance with the requirement that specialists issuing opinions should possess expert knowledge (up-to-date theoretical knowledge, deliberate practice, feedback). Given that these conditions are not met raises the question if we are even dealing with an expert. A vast majority of experts issuing opinions in autism-related cases are pediatricians, psychiatrists, and psychologists. However, in the first stages of the procedure, they are usually selected solely based on their education, and not on their practical specialization and experience (Chowaniec et al., 2005; Ericsson, 2008). The areas they represent cover an extremely wide range of potential conditions. Therefore, to perform their duties properly, each must have extensive knowledge of the condition, whether it is paranoid schizophrenia, children's depression, ADHD or autism. It is rare for one person to have such specialist knowledge. If one wants to become an expert, one cannot specialize in the entire field of psychiatry or psychology. Autism is an extremely complicated mental condition characterized by non-specific symptoms and even their absence in certain circumstances. A person who does not have extensive professional experience (deliberate practice) and in-depth theoretical knowledge may not be suitably qualified to assess the condition of a person with disabilities, even if being a licensed psychiatrist or psychologist.

Furthermore, opinions issued by medical appraisers and court experts are often authorized under conditions of strong cognitive uncertainty, which gives rise to the risk of committing an error. This is due to: (a) limited knowledge of the medical domain, (b) limited time spent on an observational survey, and (c) highly variable individual autism symptoms (often deviating from the common and the stereotypical), (d) very limited knowledge of the person examined. There is also a higher risk of being negatively influenced by heuristics and, therefore, basing the expert opinion on the existing non-rational beliefs and “myths” surrounding autism. Because of a high degree of uncertainty, psychologists and psychiatrists belong to a group of experts exposed to relatively frequent errors (Shanteau, 1992).

## Discussion: Experts are More Biased Than They May Expect

Being an expert in one area does not automatically make a person immune to non-rational beliefs from other fields of knowledge. Intuitively, it seems that experts are aware of biases in a given field of inquiry and, reasoning by analogy, should notice that similar types of biases also affect their reasoning about issues from outside their field of expertise. After all, the actual field of expertise encompasses the proper domain of highly reliable expert knowledge and a wide range of auxiliary knowledge from other disciplines with reliability diminishing proportionally to the distance from their main research area. Interestingly, at least some types of expertise could potentially produce specific types of biases, which are either extremely rare or have a low overall impact on the reasoning among the laymen.

Experts are particularly vulnerable to the blind spot bias (Pronin et al., 2002) belonging to the group of so-called egocentric biases. Generally speaking, experts are more prone to hold the conviction that they are less biased than others, including other experts, and that their field of expertise is less vulnerable to bias than other fields, which likely affects the judgments on autism as well. One of the recent studies of experts in forensic psychology provides valuable insights into the impact of the blind spot bias (Kukucka et al., 2017). A survey of 403 experienced examiners in forensic psychology from 21 countries demonstrated that experts regard their judgments as virtually infallible and, in most cases, express little appreciation for the study of cognitive biases. Moreover, most of them are of the opinion that it is possible to overcome cognitive biases by sheer willpower. Fewer than a half supported the idea of extensive blind testing in their fields, regarding it as an unnecessary and costly burden. The studies on experts’ opinions about the scope of their bias are even more insightful. 70.97% of experts in forensic psychology express the concern that forensic science as a whole is partially biased, but far less (52.36%) have the same belief about their specific field. Even fewer experts (25.69%) admit that they could be biased in their professional judgements.

Experts are also plagued by another common type of egocentric bias, which is figuratively called the illusion of control. In laypeople, the illusion of control results in a high degree of certainty that they are able to influence external events, especially preventing negative outcomes. In experts, the same mechanism causes the strong belief that they are able to reduce the extent of cognitive bias by mere willpower, which likely affects the judgments on autism However, ironically, the pursuit of active suppression of certain biases can invariably lead to more occurrences of such a bias due to the psychological mechanism known as ironic rebound or ironic processing (Wegner, 1994). For example, judges, who instruct jurors about the reliable and unreliable types of evidence usually contribute more time and effort to the explanation of how unreliable evidence biases the judgment. As a result, jurors focus more on unreliable evidence and by actively suppressing their influence on judgments, they make it more pronounced (Steblay et al., 2006). The most important brain region involved in thought suppression is supposed to be the anterior cingulate cortex, which is a part of the limbic system (Rolls, 2019). Time pressure and high cognitive load can negatively affect the thought suppression process. The ironic rebound bias is the result of two separate processes operating simultaneously, i.e. (1) operating process searching for inputs consistent with the intended mental state and (2) monitoring process searching for inputs inconsistent with the intended mental state. Under specific circumstances, such as either high cognitive load or emotional impact, the monitoring process can supersede the operating process and, as a result, put a greater emphasis on thoughts intended to be excluded from the reasoning. Medical decision-making is an obvious example of a field where time pressure is particularly high.

Another complex cognitive bias known as the curse of expertise arises when experts communicating with non-specialists subconsciously assume that both parties have the same background knowledge. However, an expert in one area is a layperson in another field. Nowadays, more and more expert judgements are the end product of consultations between many experts. As a result, the curse of expertise bias can directly affect an autism expert, who communicates with another expert from a different field during the case study. If one of them is senior, s/he can significantly underestimate the difficulties of the younger colleague (Hinds, 1999).

Also, due to strenuous mental work experts, including autism experts, are highly susceptible to mental fatigue and occupational burnout. Time pressure and dissatisfaction caused by inadequate time play a significant role. Mental fatigue can be caused by a progressive decrease in motivation-related task engagement (Gergelyfi et al., 2015). However, mental fatigue is rather difficult to assess and can vary tremendously from individual to individual. Therefore, a predefined system designed to organize experts’ activity around a particular work time and breaks schedule can be potentially ineffective due to high individual variability.

Last but not least, when considering the issue of professionals’ biases in the context of autism, it is worth mentioning the “double empathy” problem that might shed a new light on the issue (Milton, 2012). Clinical evaluations of different kinds require communication between its parties and at least some level of mutual understanding (for which comprehension of the first-person perspective is crucial). Usually, the blame for misunderstanding in the process of communication falls on the autistic person’s difficulties in social interactions. The “double empathy” theory redefines this issue by emphasizing that the problem lies on both sides, and that autistic people engage in different and not deficient ways of communication. It is also that non-autistic people have difficulties in emphatizing with and comprehending mental states of autistic people—and not merely the other way around. There is some evidence that autistic people are better at understanding and connecting with other autistic than neurotypical people (Morrison et al., 2020).Therefore, many of the professionals’ biases might be underpinned by a more general problem of incompatible “styles” of empathizing between autistic and non-autistic people, which should become a cause of reflection on the role of autistic people as experts in many clinical (and not only) situations. It is even theoretically possible that autistic medical professionals could be more impartial in their judgements concerning certain different aspects of neurodiversity, such as social anxiety or PTSD sufferers.

## How to Improve Expert Judgments on Autism

As we have shown, experts, including those working in the field of mental health (or we should say, especially those) are liable to systemic cognitive errors (biases), especially when it comes to asses highly variable individuals (like people from different end of autistic spectrum who present a completely different type of behaviour) in short time.

In contemporary research, relatively much attention is paid to the issue of accuracy and validity of diagnoses, but much less to the decisions of experts who serve on various health assessment committees, e.g. those who assess the level of a person's disability. Therefore, the following ideas are directed—but not limited to—health professionals other than diagnosticians, i.e. medical court expert witnesses or other medical assessors issuing opinions in cases concerning autistic people.

In the remainder of this paper, we introduce several suggestions for the improvement of expert judgments on autism. These suggestions are meant as solutions to problems of expert knowledge in the context of the discussed non-rational beliefs concerning autism. We can divide these strategies into self-management solutions and systemic solutions.

### Self-management Solutions

- *Mental fatigue control*. In such a specific working environment (many different/specific cases, complex area of knowledge, short time for decision, and no immediate feedback) it is crucial to control one’s fatigue level and ability to focus attention, which can be affected by intensive work or occupational burnout. One of the possible solutions to that problem can be a phone or computer application to test autism experts' level of mental fatigue and advise them to take a break at the most appropriate moment, not following a predefined schedule. For example, such an application can use the Stanford Sleepiness Scale (SSS) (Hoddes et al., 1972) as a relatively easy-to-implement mental fatigue self-report scale (Duan et al., 2018). The individually variable level of mental fatigue can provide valuable insights about the planning of work schedules.
- *Reduction of blind spot bias*. This could be done by the methods involving the subjective assessment of self-awareness, such as re-examining random particularly complicated or suspiciously straightforward cases of autism after e.g., 1 month. Time perspective and a subconscious analysis of the cases performed by the brain during leisure time can help reduce the effect of the blind spot bias. Moreover, it is important to eliminate the strong conviction about infallibility either by counseling or, at least, by self-proclamation.
  This could be also supported—at the institutional level—by administering obligatory specific intellectual humility questionnaires, which could be a helpful way to rise autism experts’ awareness of the overconfident bias (Hoyle et al., 2016).
- *Stereotypes and bias self-control*. Autism experts should be able to recognize stereotypical and non-rational beliefs concerning people they examine. O’Sullivan and Schoefield propose a general, multi-step strategy for experts for self-debiasing: Slow down; Be aware of base rates for your differentials; Consider what data is truly relevant; Actively seek alternative diagnoses; Ask questions to disprove your hypothesis; Remember you are often wrong. Consider the immediate implications of this (O’Sullivan & Schofield, 2018). Moreover, experts should self-test their awareness of general stereotypes affecting people with autism. After all, formulating an expert judgement requires a lot of biological resources. Due to the high energetical cost of constant factual and formal vigilance, the autism expert’s brain can use cheap heuristics, such as stereotypes. We speculate that when severely stressed or working under time-pressure, experts exposed to a stereotypical and often false account of autism in their private life are potentially more prone to subconsciously incorporating invalid stereotypes into their judgments.
- *Ironic processing control*. This solution involves measures taken to diminish the effect of ironic processing and, as a result, the illusion of control bias. One of the relatively uncomplicated methods is the direct exposition, i.e. the meeting between an expert and an autistic person being examined to prevent reification of the person and analysis founded upon the stack of paperwork alone. The face-to-face meeting can be a sufficient stimulus for the operating process to supersede the monitoring process and, as a result, to prevent the ironic rebound effect. Experts can be safely instructed about various types of cognitive biases without the fear of ironic rebound. This point is especially important today due to the COVID-19 pandemic, when autism health assessments are conducted on-line or even entirely *in absentia*. Such a situation additionally detaches an expert from an autistic person examined, forcing an ironic processing effect.

This is also an interesting challenge at the systemic/institutional level. We can imagine that the whole process of health assessment and an autistic person’s presentation could be changed to ensure that an expert could see such person holistically, with his/her life story, particular difficulties, in the context of a whole social situation, and not merely as a “bag of symptoms” or a “set of medical files” (Table 2).

**Table 2:** Strategies for the improvement of expert judgments

Self-management solutions	
Mental fatigue control	Work schedule planned individually with mobile/computer application
Reduction of blind spot bias	Re-examining random particularly problematic and suspiciously straightforward cases of autism
Stereotypes and bias self-control	Self-testing for autism stereotypes awareness and multi-step self-debiasing strategies
Ironic processing control	Direct meetings between an expert and an autistic person being examined

Systemic solutions	
General debiasing training	Mandatory training for court and medical experts on autism including 3 perspectives: virtue theory, epistemic paternalist theory, collectivist theory
Competencies control	Precise control of autism experts’ formal qualifications and practical experience
Different perspectives	Taking into account all available perspectives: first- and third-person testimonies, medical documentation etc
Feedback	Introducing cross-checking of decisions between the teams of experts or systemic oversight of the correctness of opinions by relevant external and independent institutions

### Systemic Solutions

- *General debiasing training*. The first proposed systemic solution is to introduce mandatory training for court and medical experts assessing autistic people on the impact of stereotypes and non-rational beliefs on their cognitive disposition and therefore on their ability to make correct decisions. Raising awareness about this impact is a preliminary step of its reduction. Such training programs should draw on at least three perspectives on potential bias sources: virtue theory (biases can be controlled by e.g. rising epistemic/intellectual humility or self-vigilance attitude), epistemic paternalist theory (biases can be controlled by “identifying and manipulating the situational factors that bias our cognition”) and collectivist theory (biases can be controlled by a collective, social discussion) (Bland, 2020).
- *Competencies control*. The second solution is to formally specify the specialisation of court experts. Even a doctor specialising in child psychiatry is unable to possess the knowledge necessary to issue a credible opinion concerning every mental state, disorder or illness recognised in medical classifications. Court experts databases should also include the practical deliberate experience of each expert (Ericsson, 2008). Taking these criteria into account would increase the chances of an appropriate expert being selected for the given case and thus lowers the chances of an unreliable opinion being issued, one caused by insufficient familiarity with the domain in which the evaluation is performed.
  This solution is connected with the need of introducing some means of verification of the actual (and not only formal) competencies of autism experts seeking to become court or other medical assessors, such as the ones functioning in France. Aside from the currently required proof of having appropriate education, the candidate for such an expert should participate in appropriate workshops, conferences or publish in specialist scientific journals in order to confirm regularly supplementing knowledge with the latest scientific findings.
- *Different perspectives*. Lenart and Pasternak suggest that proper autism diagnosis (also applies to other medical assessments, especially in mental health area) should include four different perspectives: “(a) a viewpoint related to general condition of the child’s health; (b) nosological, psychometric and functional perspective; (c) systemic and social perspective, making a real attempt to understand symptoms and underlying calming and exacerbating factors in the context of family and social setting; (d) emphasizing resources and other areas related to health” (Lenart & Pasternak, 2021). This should include both first-person (or family’s) perspective statements and the full scope of autistic person’s medical documentation. Also, the work of interdisciplinary teams of professionals cannot be overestimated in the full diagnostic process. Different points of view of experts representing different scientific fields may shed important light on observed symptoms and their causes and result in differentiated, well- suited supporting programs (LeMay et al., 2019). Yet, quite frequently the discussed disability assessments are very short (lasting several to a dozen or so minutes, whereas a solid autism diagnosis typically requires several hours of observations). Such brief evaluations, which experts often perform today with insufficient competencies, should be combined with analysis of a person’s medical documentation following appropriate procedures. This documentation, drafted by a team of specialists who work with a specific autistic person on a daily basis, constitutes a much more extensive material for analysis than an observation that lasts mere several minutes. Unfortunately, court experts and medical assessors often ignore or question opinions issued by specialists taking care of the particular autistic person on a daily basis. Furthermore, a study by Morrisson et al. on the issue of the “double empathy” problem, shows that autistic people might better cope with understanding and connecting with other autistics than non-autistic people do (Morrison et al., 2020). Therefore supporting autistic people in becoming professionals working as experts in such contexts might lead to better understanding of parties and to reducing professional bias.
- *Feedback*. As Klein’s, Simon’s, Kahneman’s and Gigerenzer’s research shows, proper feedback information is a crucial element of the decision-making practice and its evaluation. Unfortunately, feedback received by medical examiners is minimal despite the fact that they usually act as the final decision-makers. Some persistent autistic people or their parents may decide to enforce their rights in higher institutions by appealing to better-qualified expert teams. However, this is not a common practice as it is complicated and time-consuming.

It is worth adding that research on natural decision-making theory concerned a group of people frequently performing more or less the same activity. Chess games differ from one another but follow the same rules. Firefighters, who intuitively, and thus without an extended logical analysis, were able to determine the course of events during operation (e.g., when to leave a building to avoid danger), also acted in a rather specific problem field. Moreover, both of these groups received an immediate response as to whether their decision was appropriate. When their move turned out to be correct or the building collapsed, the decision-maker had an easily noticeable criterion for the correctness or wrongness of his decision. The opportunity to receive such feedback is one of the key elements in improving autism experts’ expertise.

The most difficult element to comply with among the proposed solutions is obtaining feedback of appropriate quality on the decisions issued. This could be resolved by cross-checking decisions between the teams of experts or systemic oversight of the correctness of opinions by relevant external and independent institutions, which could verify their accuracy.

Last but not least, we would like to draw attention to the issue of more general nature. As it is well known, not every autistic person accepts any of the medical/disability labels. Many refuse to see themselves and build their identities on the basis of the biomedical perspective that they consider stigmatizing. Therefore, activists, scientific and government-related communities should constantly discuss and try to improve the system of granting public support to autistic (and not only) people, to make it more accessible, more efficient, and less stigmatizing. Instead of operating with readily available labels (ASD, bipolar disorder, etc.), fixed therapeutic programs or other ways of support, we suggest to focus on functional and ecological diagnoses/assessment, which could show individual difficulties of each person in different contexts, and therefore allow for taylored supporting solutions devoid of stigmatizing labels.

Interestingly, in the Polish health care system, there is an equivalent of such a solution in the case of the youngest children, known as the Early Development Support system. Unfortunately, it covers only children up to 6 years of age and very often does not function in accordance with its assumptions. Hence, the recommendations and forms of support required by a given person are not implemented.

Yet, as always with mass-scale issues and systems, such change needs much more resources (money, time, appropriately educated staff, etc.) and is always associated with balancing between the needs of individuals and the requirements of large scale, systemic data and resources management.

## Footnotes

1 Currently, there is no clear and consistent consensus across the autistic community on the terminology appropriate to describe autism. We believe that, above all, the choice of a particular option (e.g. autistic person vs. person with autism) should depend on the individual preferences of the person, to whom the terminology used applies. However, for the purposes of this paper, we have decided to consistently use the term “autistic” instead of “on the spectrum” and “autism” instead of “Autism Spectrum Disorder”. This choice of terminology is supported by the opinions of autism community (Kenny, 2016).

2 https://committees.parliament.uk/committee/164/work-and-pensions-committee/news/159277/experts-questioned-on-flaws-in-assessments-for-disability-and-healthrelated-benefits/; https://committees.parliament.uk/call-for-evidence/601; https://committees.parliament.uk/work/1468/health-assessments-for-benefits/.
